# Synthesis, Molecular Docking, Molecular Dynamic Simulation Studies, and Antitubercular Activity Evaluation of Substituted Benzimidazole Derivatives

**DOI:** 10.1155/2024/9986613

**Published:** 2024-03-28

**Authors:** Shankar Thapa, Mahalakshmi Suresha Biradar, Shachindra L. Nargund, Iqrar Ahmad, Mohit Agrawal, Harun Patel, Ashish Lamsal

**Affiliations:** ^1^Department of Pharmacy, Universal College of Medical Sciences, Bhairahawa 32900, Nepal; ^2^Department of Pharmaceutical Chemistry, Nargund College of Pharmacy, Bengaluru 560085, Karnataka, India; ^3^Department of Pharmacy, Madan Bhandari Academy of Health Sciences, Hetauda, Nepal; ^4^Department of Pharmaceutical Chemistry, Al-Ameen College of Pharmacy, Bengaluru 560027, Karnataka, India; ^5^Department of Pharmaceutical Chemistry, Prof. Ravindra Nikam College of Pharmacy, Gondur, Dhule 424002, Maharashtra, India; ^6^School of Medical & Allied Sciences, K.R. Mangalam University, Gurugram, Haryana, India; ^7^Division of Computer Aided Drug Design, Department of Pharmaceutical Chemistry, R. C. Patel Institute of Pharmaceutical Education and Research, Shirpur 425405, Maharashtra, India

## Abstract

Tuberculosis, also known as TB, is a widespread bacterial infection that remains a significant global health issue. This study focuses on conducting a thorough investigation into the synthesis, evaluation of anti-Tb activity, molecular docking, and molecular dynamic simulation of substituted benzimidazole derivatives. A series of twelve substituted benzimidazole derivatives (**1**–**12**) were successfully synthesized, employing a scaffold consisting of electron-withdrawing and electron-donating groups. The newly synthesized compounds were defined by their FTIR, ^1^H NMR, and mass spectra. The microplate Alamar blue assay (MABA) was used to evaluate the antimycobacterial activity of the synthesized compound against *Mycobacterium tuberculosis* (Mtb). Compounds **7** (MIC = 0.8 g/mL) and **8** (MIC = 0.8 g/mL) demonstrated exceptional potential to inhibit *M. tuberculosis* compared to the standard drug (isoniazid). In addition, the synthesized compounds were docked with the Mtb KasA protein (PDB ID: 6P9K), and the results of molecular docking and molecular dynamic simulation confirmed the experimental results, as compounds **7** and **8** exhibited the highest binding energy of −7.36 and −7.17 kcal/mol, respectively. The simulation results such as the RMSD value, RMSF value, radius of gyration, and hydrogen bond analysis illustrated the optimum potential of compounds **7** and **8** to inhibit the *M. tuberculosis* strain. Hydrogen bond analysis suggested that compound **7** has greater stability and affinity towards the KasA protein compared to compound **8**. Moreover, both compounds (**7** and **8**) were safe for acute inhalation and cutaneous sensitization. These two compounds have the potential to be potent *M. tuberculosis* inhibitors.

## 1. Introduction

The infectious disease tuberculosis (TB) is caused by the bacterium *Mycobacterium tuberculosis* (Mtb) [1]. It is the 13^th^ leading cause of death globally, accounting for 1.6 million fatalities in 2022 alone [2]. The antimicrobial agents such as first-line drugs (such as isoniazid, rifampin, and pyrazinamide) and second-line drugs (such as aminoglycosides and fluoroquinolones) are one of the primary strategies for treating tuberculosis [3]. The emergence of drug-resistant Mtb strains has posed a significant obstacle to the treatment of the disease. Resistance to anti-TB medications is a significant concern in the treatment of tuberculosis. The situation has become more complex and costly due to the emergence of multidrug-resistant (MDR) tuberculosis, posing additional challenges to therapy. Therefore, new medicines with a low potential for resistance must be developed [4].

**Figure 1:**
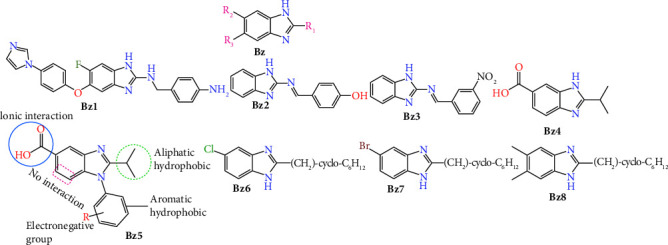
Benzimidazole basic moiety and published benzimidazole derivatives against antibacterial drug design and 3D QSAR approach of design. “**Bz**” represents benzimidazole and it is given to code the compound.

**Figure 2:**
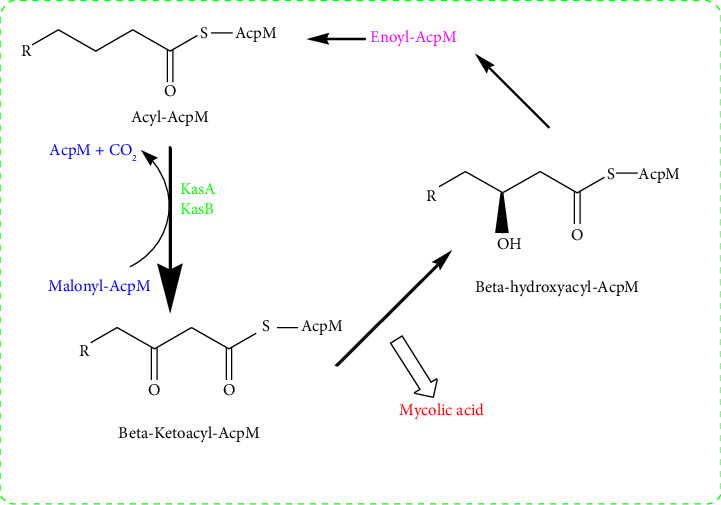
Role of KasA and KasB in mycolic acid biosynthesis [8].

In synthetic chemistry, two prevalent strategies for drug design involve the repurposing of an existing parent molecule to enhance its activity, as well as the development and synthesis of a novel therapeutic molecule that has potential activity. The first strategy has a high success rate, and the benzimidazole scaffold (Figure 1, **Bz**) may be the best candidate to modify into prospective anti-TB agents from this strategy [5, 6]. Benzimidazoles are heterocyclic compounds containing an atom of nitrogen in the ring system. They have a wide variety of biological actions, including anti-inflammatory, antitumor, antibacterial, and antiparasitic effects [7]. The ability of substituted benzimidazoles to inhibit the synthesis of mycolic acids, a key component of the cell wall of *M. tuberculosis*, is one of their primary mechanisms of action. Mycolic acids are essential for the survival and persistence of Mtb, and their synthesis is mediated by the mycolic acid synthase group of enzymes. Specifically, KasA, which is responsible for the elongation of the fatty acid moiety, facilitates the synthesis of mycolic acid. Moreover, KasA and KasB add two carbons to acyl-AcpM (mycobacterial acyl carrier protein, AcpM) to synthesize ketoacyl-AcpM (Figure 2) although KasA is superior to KasB due to its independence from cultural requirements (nature of genetic disruption growth media). Therefore, KasA protein is chosen over KasB for molecular docking investigations [9].

Substituted benzimidazoles are believed to compromise the bacterial cell wall and cause its mortality. Benzimidazoles may also destroy bacterial cells by competing with purines and inhibiting the formation of nucleic acids and proteins, according to a second hypothesis regarding their mode of action. Moreover, it has been reported that these compounds are potent topoisomerase inhibitors [10]. Developing novel agents with minimal risk of resistance is crucial for the prevention of TB. The chemical space of benzimidazole with aromatic substitution greatly enhances the binding affinity to the novel tubercular receptor. The basic benzimidazole moiety shows significant interactive property but to stabilize the interaction, substitution on C2 position is necessary (Figure 1, compound **Bz1**). This combination results the new antitubercular compound along with stability [11]. The substitution of substituted aryl methananimine ring on C2 position of benzimidazole moiety (Figure 1, compound **Bz2** and **Bz3**) reports the excellence antibacterial activity and their molecular shows the DNA gyrase inhibition as their major mode of action [12]. The antitubercular action of synthesized 1,2-disubstituted benzimidazole-5-carboxylic acid derivatives is predicted by the 3D QSAR (quantitative structure activity relationship) model, which also provides a MIC value of 0.0975 *μ*M (**Bz4** and **Bz5**). Compounds (**Bz6**, **Bz7**, and **Bz8**) with a cyclohexylethyl substituent at the C2 position and a halogen atom or methyl group at the benzimidazole ring can increase the compound's overall antitubercular efficacy [13, 14]. Several studies also indicate that the electron-withdrawing group can serve as a promising substitution in benzimidazole scaffolds to boost biological activity [15]. Moreover, addition of heterocyclic and aromatic substitution on benzimidazole leads to structural flexibility and target specificity [16].

The goal of this research is to synthesize a series of substituted benzimidazole compounds and assess their antituberculosis activity. Molecular modelling was used to provide insight into the binding mechanisms at molecular level. The toxicity study is also conducted to determine the safety range of synthesized compounds. These compounds have the potential to enhance the treatment of tuberculosis and resolve the problem of drug-resistant *M. tuberculosis* strains.

## 2. Materials and Methods

### 2.1. Reagents and Instruments

All the solvent and reagents used in the experiment were bought from a commercial source (analytical grade, IR grade). Shimadzu-5400 FTIR (Fourier's transform infrared) spectroscopy was used to record the IR spectra, while GC-MS (gas chromatography-mass spectroscopy) (from SAIF IIT-Madrs, India) was used to record the mass spectra (for basic molecule only). With a Bruker Advance-400 MH_Z_ spectrometer, ^1^H NMR (proton nuclear magnetic resonance) was recorded (solutions in DMSO or CDCl_3_). The chemical shift values were given in, parts per million (ppm), about the internal standard. To check the completion of reactions, 0.2 mm silica gel 60 F254 precoated (E-Merck) plates were used for thin-layer chromatographic (TLC) studies. Open glass capillaries were used to calculate the uncorrected melting points (±2°C).

### 2.2. Basic Compound Synthesis

**Figure 3:**
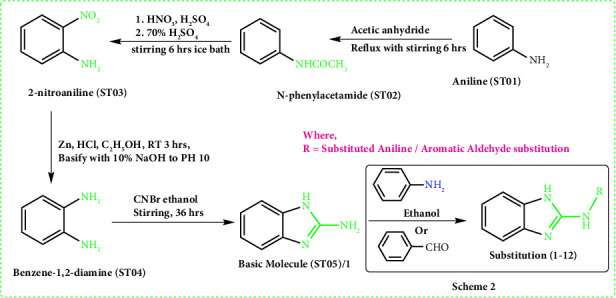
Synthetic scheme 1 for basic molecule (**ST05**) and brief of scheme 2.

The basic molecule **(ST05)** was synthesized by slightly modifying the previously published procedure [17]. To protect the amine group of aniline **(ST01)** during nitration, the concept of protecting group was employed. Reduction of 2-nitroanioline (**ST03**) led to the synthesis of benzene-1,2-diamine **(ST04)**. By cyclizing the diamino compound with cyanogen bromide, the basic 2-amino benzimidazole ring **(ST05)** was synthesized (Figure 3). Melting point determination, TLC, FTIR spectroscopy, ^1^H NMR, and mass spectroscopy (only for compound **1**) were used to characterize the synthesized compounds (Supplementary material Figure S1–S3).

### 2.3. Synthesis of Derivatives

**Figure 4:**
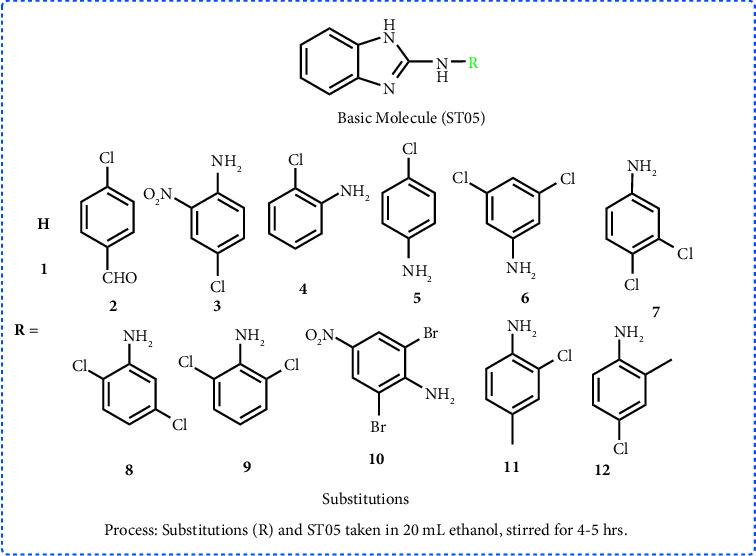
Detailed synthetic scheme 2 for the synthesis of benzimidazole derivatives (**1**–**12**).

The procedure for amine substitution in **ST05** was derived from previously published work by R. Kalarani and colleagues (Figure 4). Approximately, 6 mmol of **ST05** and equimolar of aldehyde or amine derivatives were added to 20 mL of ethanol and agitated for four to eight hours until a yellow precipitate was formed [18]. It was filtered, ethanol washed, and desiccated for 12 hours. From ethanol, recrystallization was performed and TLC was used to verify the purity. The yield ranged from 45 to 78%. The FTIR, ^1^H NMR, and mass spectra of derivatives compounds (**1–12**) are given in Supplementary material (Figure S4–S28).

### 2.4. Anti-TB Activity Using the Microplate Alamar Blue Assay (MABA) Method

Using the BACTEC radiometric method, the MABA test was conducted in a controlled laboratory environment to ascertain the efficacy of synthesized benzimidazole derivatives against *M. tuberculosis*. Aliquot 100 *μ*L of Middlebrook 7H9 broth was applied to each well of a 96-well plate initially. Then, a series of benzimidazole derivatives dilutions in Middlebrook 7H9 broth, ranging from 100 to 0.2 g/mL, were prepared and added to the designated wells. Subsequently, 25 *μ*L of a freshly prepared 1 : 1 mixture of 10% Tween-80 and Alamar Blue reagent was added to each well, followed by gentle agitation. Covering the plate and incubating it at 37°C for 24–48 hours. The plate was then inserted into the BACTEC radiometric apparatus, which measured the growth index (GI) of each well following incubation. The GI values were recorded and used to determine the sample's minimum inhibitory concentration (MIC) against *M. tuberculosis*. The MIC was determined by identifying the maximum sample dilution at which minimal or no growth was observed. To ensure reproducibility, the experiment was repeated three times, and the MIC values obtained were averaged. The results were then analyzed and interpreted based on established MIC breakpoints for a sample and *M. tuberculosis* susceptibility, ensuring compliance with biosafety guidelines throughout the entire procedure [19, 20].

Standard strain used: *Mycobacteria tuberculosis* (H37 RV strain): ATCC no. 27294.

Reference drugs used: isoniazid, ethambutol, pyrazinamide, rifampicin, and streptomycin.

### 2.5. Molecular Docking

The synthesized benzimidazole compounds were drawn in Chem Draw in structural data file (SDF) format and subjected to ligand preparation using the ligand prep module of the Schrodinger suite. Using default conditions at pH 7.0 ± 2.0, the ligand preparation preserves chirality and generates at least five low-energy stereoisomers per ligand [21, 22]. Using Maestro's protein preparation wizard, the crystal structure of *M. tuberculosis* (Mtb) KasA in complex with DG167 (PDB ID: 6P9K) was generated. This included the addition and optimization of hydrogen bonds, the elimination of atomic collisions, the addition of formal charges to the hetero groups, and the final optimization at neutral pH. Using the OPLS-3e (optimized potential for liquid simulations-3e) force field, the structure was then reduced. A three-dimensional receptor interaction grid was constructed at the center of the bound ligand (DG167). Finally, docking was accomplished using the SP docking technique [23, 24].

### 2.6. Molecular Dynamic (MD) Simulation

“Schrodinger Desmond MD simulation software (version 2021-1) was installed on a Z4 HP workstation with the configuration Ubuntu 22.04.2 LTS 64-bit, Intel Xeon W-2245 @ 3.90 GHz, 8-Cores, CUDA 12, and NVIDIA RTX A4000 graphics processing unit to conduct MD studies for the lowest docking pose” [25] of promising compounds **7** and **8**. To solvate the systems, a simple point charge (SPC) water model was utilized, and a suitable pair of ions, such as sodium and chloride, were used to establish a neutral environment. Using the Desmond System Builder salt concentration panel, 0.15 M NaCl was added to the physiological system. This solvated and neutral system was subjected to unrestricted energy reduction using the steepest descent criterion in the OPLS3e force field to resolve steric conflicts [26]. As the same parameters were used for the systems analyzed in this research, previous research may contain additional information regarding the MD (simulation box type, thermostat, barometer values, calculations of short- and long-range interactions, etc.) [27].

### 2.7. Toxicity Prediction

STopTox server (https://stoptox.mmL.unc.edu/) predicted the fragment contributing to increased or decreased acute inhalation toxicity and cutaneous sensitization for compounds **7** and **8** [28].

## 3. Results and Discussion

### 3.1. Chemistry

It is essential to evaluate the chemical stability, reactivity, and solubility profile, as well as the biological activity, of a substituted benzimidazole using a variety of techniques to determine how it could be used as a therapeutic agent or for other purposes. The substitution of electron-donating or electron-withdrawing groups on benzimidazole can significantly alter its biological activity. The chemical stability, reactivity, and/or solubility of the molecule may be impacted by substitutions at the different position on ring, which may then have an impact on the molecule's capacity to bind to a particular target or traverse a particular biological membrane, among other properties. The presence of electron-withdrawing groups, such as chlorine or nitro, can increase the stability and hydrophobicity of a molecule. This can make the molecule more selective for a specific target and increase its potency by decreasing its metabolism or elimination rate. The electron-withdrawing groups may also enhance the lipophilicity of the molecule, thereby facilitating its passage through cell membranes. This can be advantageous for drug molecules, enhancing their therapeutic efficacy [29, 30].

In contrast, electron-donating groups such as amino or hydroxyl groups can decrease the molecule's stability and increase its hydrophilicity. This can reduce the selectivity and potency of the molecule by accelerating its metabolism or elimination from the body, and it may also have difficulty traversing cell membranes [31, 32]. Benzimidazole's biological activity can also be affected by the position of the substituent group. A substituent group at the C2 position of the ring can interact with the nitrogen atoms at positions 1 and 3 and may have a greater influence on the molecule's chemical properties than a substituent group at a different position [14].

### 3.2. Spectroscopy Results

The FTIR, ^1^H NMR, and mass spectra of derivatives compounds (**1**–**12**) are given in Supplementary material (Figure S4–S28).

*1H-1,3-benzodiazol-2-amine* (**1**). White powder. Yield: 60%; MP: 180–182°C; *R*_*f*_: 0.76 (ethyl acetate: hexane, 3 : 1); FTIR (KBr, cm^−1^):, 3429 cm^−1^ (N-H primary amine str), 3371 cm^−1^ (-NH str secondary amine), 3140 cm^−1^ (-CH str heterocyclic ring), 2945 cm^−1^ (-CH str aromatic), 1700 cm^−1^(C=C str aromatic), 1697 cm^−1^(N=C str); ^1^H NMR (400 MHz, DMSO) *δ* ppm 6.36–7.11 (m, 4H, aromatic‐H), 11.53 (s, 2H, amine-NH_2_); GCMS, m/z = 133 (M + H)^+^.

*4-[(1H-1,3-benzodiazol-2-yl) amino] benzaldehyde* (**2**). Slightly white solid. Yield: 51%; MP: 220x222°C; *R*_*f*_: 0.68 (ethyl acetate: hexane, 3 : 1); FTIR (KBr, cm^−1^): 3445 cm^−1^(N-H str. primary amine), 3064 cm^−1^ (C-H str. heterocyclic ring), 2922 cm^−1^ (-CH str. aromatic), 1744 cm^−1^ (CHO str.), 1611 cm^−1^ (C=C str. aromatic), 1430 cm^−1^ (C=O str.); ^1^H NMR (400 MHz, DMSO) *δ* ppm 7.21(d, 2H, aromatic‐H, *J* = 7 Hz), 7.45 (d, 2H, aromatic‐H, *J* = 7 Hz), 8.09–8.20 (m, 4H, aromatic-H), 12.74 (s, 1H, aldehyde-H).

*N1-(1H-1,3-benzodiazol-2-yl)-3-nitrobenzene-1,4-diamine* (**3**). Yield: 52%; MP: 132–134°C; *R*_*f*_: 0.91; FTIR (KBr, cm^−1^): 3466 cm^−1^ (-NH str.), 3354 cm^−1^ (-NH_2_ str.), 2922 cm^−1^ (C-H aromatic), 1626 cm^−1^ (C=C str aromatic), 1543 cm^−1^ (-NO_2_ asy.), 1336 cm^−1^ (-NO_2_ sym.); ^1^H NMR (400 MHz, DMSO) *δ* ppm 6.54–6.85 (d, 3H, nitroaniline ring, aromatic‐H, *J* = 6 Hz), 7.00–7.09 (m, 4H, benzimidazole ring aromatic-H), 10.64 (s, 2H, amine-NH_2_).

*N1-(1H-1,3-benzodiazol-2-yl) benzene-1,2-diamine* (**4**). Yield: 60%; MP: 200–202°C; *R*_*f*_: 0.32 (ethyl acetate: hexane, 3 : 1); FTIR (KBr, cm^−1^): 3385 cm^−1^ (-NH_2_ str.), 3066 cm^−1^ (C-H str. aromatic), 1652 cm^−1^ (C=C str. aromatic); ^1^H NMR (400 MHz, DMSO) *δ* ppm 6.37–6.85 (m, 4H, aniline ring, aromatic‐H), 7.07–7.10 (m, 4H, benzimidazole ring aromatic-H), 10.68 (s, 2H, amine-NH_2_).

*N1-(1H-1,3-benzodiazol-2-yl) benzene-1,4-diamine* (**5**). Yield: 74%; MP: 90–92°C; *R*_*f*_: 0.84 (ethyl acetate: hexane, 3 : 1); FTIR (KBr, cm^−1^): 3383 cm^−1^ (-NH_2_ str.), 2924 cm^−1^ (C-H aromatic), 1639 cm^−1^ (C=C str. aromatic); ^1^H NMR (400 MHz, DMSO) *δ* ppm 6.38–7.00 (m, 4H, benzimidazole ring aromatic-H), 7.02–7.84 (m, 4H, aniline ring, aromatic‐H), 10.64 (s, 2H, amine-NH_2_).

*N1-(1H-1,3-benzodiazol-2-yl)-5-chlorobenzene-1,3-diamine* (**6**). Yield: 58%; MP: 170–172°C; *R*_*f*_: 0.74 (ethyl acetate: hexane, 3 : 1); FTIR (KBr, cm^−1^): 3385 cm^−1^ (-NH_2_ str.), 3090 cm^−1^ (C-H str. heterocyclic ring), 1872 cm^−1^(C=C str. aromatic), 1624 cm^−1^(N=C str.), 798.6 cm^−1^ (C-Cl str.); ^1^H NMR (400 MHz, DMSO) *δ* ppm 6.54–6.85 (m, 3H, chlorobenzene ring, aromatic‐H), 7.07–7.10 (m, 4H, benzimidazole ring aromatic-H), 10.72 (s, ^1^H, amine-NH_2_).

*N1-(1H-1,3-benzodiazol-2-yl)-6-chlorobenzene-1,3-diamine* (**7**). Yield: 51%; MP: 56–58°C; *R*_*f*_: 0.50 (ethyl acetate: hexane, 3 : 1); FTIR (KBr, cm^−1^): 3417 cm^−1^ (NH_2_ str.), 3321 cm^−1^(-NH str.), 3090 cm^−1^ (C-H str. heterocyclic ring), 1872 cm^−1^(C=C str. aromatic), 1624 cm^−1^(N=C str.), 819 cm^−1^(C-Cl str.); ^1^H NMR (400 MHz, DMSO) *δ* ppm 6.84–7.20 (m, 4H, benzimidazole ring aromatic-H), 7.38–7.50 (m, 3H, chlorobenzene ring, aromatic‐H), 10.66 (s, 2H, amine-NH_2_).

*N1-(1H-1,3-benzodiazol-2-yl)-4-chlorobenzene-1,3-diamine* (**8**). Yield: 72%; MP: 120–122°C; *R*_*f*_: 0.83 (ethyl acetate: hexane, 3 : 1); FTIR (KBr, cm^−1^): 3385 cm^−1^ (NH_2_ str.), 3321 cm^−1^ (NH str.), 2924 cm^−1^ (C-H aromatic), 1631 cm^−1^ (C=C str aromatic), 819 cm^−1^ (C-Cl); ^1^H NMR (400 MHz, DMSO) *δ* ppm 6.53–6.85 (m, 4H, benzodiazole ring aromatic-H), 7.08–7.20 (m, 3H, chlorobenzene ring, aromatic‐H), 10.58 (s, 2H, amine-NH_2_).

*N1-(1H-1,3-benzodiazol-2-yl)-3-chlorobenzene-1,2-diamine* (**9**). Yield: 56%; MP: 95–97°C; *R*_*f*_: 0.75 (ethyl acetate: hexane, 3 : 1); FT-IR (KBr, cm^−1^): 3387 cm^−1^ (NH_2_ str.), 2922 cm^−1^ (C-H aromatic), 1649 cm^−1^ (C=C str. aromatic), 896 cm^−1^ (C-Cl str.); ^1^H NMR (400 MHz, DMSO) *δ* ppm 6.35–6.82 (m, 4H, benzimidazole ring aromatic-H), 7.70–7.78 (m, 3H, chloro aniline ring, aromatic‐H), 10.69 (s, 2H, amine-NH_2_).

*N1-(1H-1,3-benzodiazol-2-yl)-3-bromo-5-nitrobenzene-1,2-diamine* (**10**). Yield: 56%; MP: 230–232°C; *R*_*f*_: 0.82 (ethyl acetate: hexane. 3 : 1); FTIR (KBr, cm^−1^): 3370 cm^−1^ (NH_2_ str.), 3096 cm^−1^ (-NH str.), 2916 cm^−1^ (C-H aromatic), 1626 cm^−1^ (C=C str aromatic), 1543 cm^−1^ (NO_2_ asy.), 1462 cm^−1^ (-NO_2_ sym.), 862 cm^−1^ (C-Br); ^1^H NMR (400 MHz, DMSO) *δ* ppm 7.40–7.55 (m, 4H, benzodiazole ring aromatic-H), 7.78 (d, 2H, bromo aniline ring, aromatic‐H, *J* = 8 Hz), 10.69 (s, 2H, amine-NH_2_).

*N1-(1H-1,3-benzodiazol-2-yl)-5-methylbenzene-1,2-diamine* (**11**). Yield: 51%; MP: 226–228°C; *R*_*f*_: 0.42 (ethyl acetate: hexane, 3 : 1); FTIR (KBr, cm^−1^): 3385 cm^−1^ (-NH_2_ str.), 3097 cm^−1^ (C-H str heterocyclic ring), 3082 cm^−1^ (C-H aromatic), 1651 cm^−1^ (C=C str. aromatic), 1564 cm^−1^ (N=C str.), 896 cm^−1^ (C-Cl str.); ^1^H NMR (400 MHz, DMSO) *δ* ppm 2.10 (s, 3H, -CH_3_), 6.50–7.21 (m, 4H, benzimidazole ring aromatic-H), 7.45–7.68 (m, 3H, aniline ring, aromatic‐H), 10.67 (s, 2H, amine-NH_2_). *N1-(1H-1,3-benzodiazol-2-yl)-3-methylbenzene-1,4-diamine* (**12**). Yield: 52%; MP: 234–236°C; *R*_*f*_: 0.86 (ethyl acetate: hexane, 3 : 1); FTIR (KBr, cm^−1^): 3389 cm^−1^ (NH2 str.), 3064 cm^−1^ (C-H str. heterocyclic ring), 2928 cm^−1^ (C-H aromatic), 1654 cm^−1^ (C=C str aromatic), 1564 cm^−1^ (N=C str.), 1280 cm^−1^ (C-F), 1267 cm^−1^ (C-O str.); ^1^H NMR (400 MHz, DMSO) *δ* ppm 3.10 (s, 3H, -CH_3_), 6.10–6.84 (m, 4H,benzodiazole ring aromatic-H), 7.17–7.50 (m, 3H, aniline ring, aromatic‐H), 10.66 (s, 2H, amine-NH_2_).

Where, *s* = singlet, *m* = multiplet, str. = stretching, syn. = symmetric, asy. = asymmetric.

### 3.3. Evaluation of Anti-TB Activity

Minimum inhibitory concentration (MIC) values pertain to the efficacy of various compounds against *M. tuberculosis* isolates. The MIC values are measured in micrograms per milliliter (*μ*g/mL) and indicate the lowest concentration of a compound that can inhibit bacterial growth. Compounds **7** and **8** have MIC values of 0.8 *μ*g/mL, which are lower than the MIC values of standard drug isoniazid (MIC = 1.6 *μ*g/mL), pyrazinamide (MIC = 3.12 *μ*g/mL), and ethambutol (MIC = 1.6 *μ*g/mL). This indicates that compounds **7** and **8** may be more effective against Mtb than the conventional drugs isoniazid, pyrazinamide, and ethambutol. Compounds **7** and **8** have MIC values of 0.8 *μ*g/mL, which is equivalent to the MIC values of the conventional antibiotic rifampicin and streptomycin (MIC = 0.8 *μ*g/mL). The result indicates that compounds **7** and **8** are equally effective against the tested microorganisms such as rifampicin and streptomycin. In contrast, compound **2** has a MIC of 50 *μ*g/mL, which is significantly higher than the MICs of all five conventional medications. This indicates that compound **2** is less efficacious than standard anti-TB medications. Compounds **4, 5, 10, 11,** and **12** have MIC values of 6.26 *μ*g/mL, which is greater than the MIC values of the standard antibiotic isoniazid (MIC = 1.6 *μ*g/mL), ethambutol (MIC = 1.6 *μ*g/mL), rifampicin (MIC = 0.8 *μ*g/mL), and streptomycin (MIC = 0.8 *μ*g/mL). The compounds **3**, **6**, and **9** have similar MIC values as that of isoniazid, i.e., 1.6 *μ*g/mL. This indicates that compounds **3**, **6**, and **9** are equally efficacious against Mtb as isoniazid. All the information is presented in Table 1.

**Figure 5:**
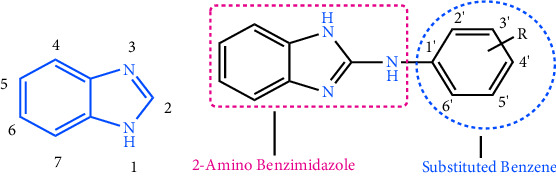
Basic benzimidazole structure (a) and diagrammatic representation of synthesized derivatives (b).

Depending on where the substitution occurs on the benzene ring, a molecule's activity may change. The charge on the moiety is stabilised in compounds **7** and **8** due to the presence of electron-donating and electron-withdrawing groups in the appropriate places (Figure 4), which may account for their enhanced activity. Both 2′ and 4′ (**7**) or 3′ and 4′ (**8**) (Figure 5) were shown to be hotspots of activity. The MIC for compounds **11** and **12** is 6.25 *μ*g/mL because they include a strong electron-donating group (-NH_2_) and a mild electron-donating group (-CH_3_) in the benzene ring. The -CHO substitution at position 4′ (**2**) on the benzene ring, on the other hand, completely abolishes activity (Figure 5). The binding affinity of the compounds to their target may change as a result of this spatial variation.

**Figure 6:**
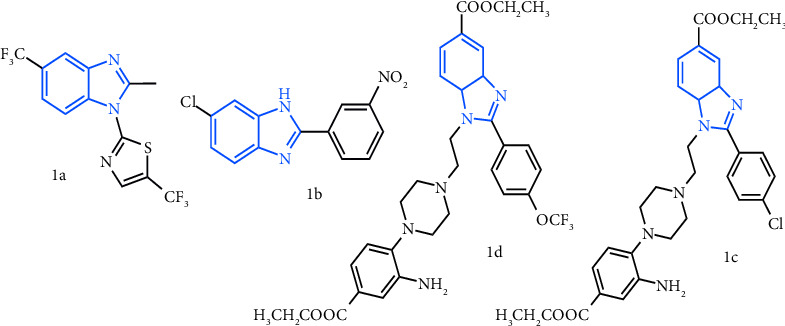
Some published promising anti-TB compounds. Blue color indicates the benzimidazole moiety.

Several substituted benzimidazoles have been evaluated in preclinical studies for their anti-TB activity. Veena et al. developed **1a** (Figure 6), one of the most promising trisubstituted benzimidazoles that have shown promise to suppress the proliferation of Mtb. It is a derivative of benzimidazole moiety whose potency (MIC = 0.28 *μ*M) and pharmacokinetic properties have been modified to obtained better therapeutic outcomes [33]. Similarly, **1b**, which was developed by Jiménez-Juárez et al., is among the most promising compounds. In in vitro and in vivo investigations, the disubstituted benzimidazole **1b** exhibited potent activity against Mtb. It is extremely selective for Mtb and has a low resistance potential. In animal studies, it has also demonstrated adequate oral bioavailability and a favorable safety profile [34]. Yoon et al. reported that compounds **1c** and **1d** showed excellent potency against Mtb-H37RV with MIC of <0.2 *μ*M [35]. In addition, several substituted benzimidazoles have demonstrated favorable oral bioavailability and safety profiles based on in silico studies [36].

### 3.4. Toxicity Prediction

**Figure 7:**
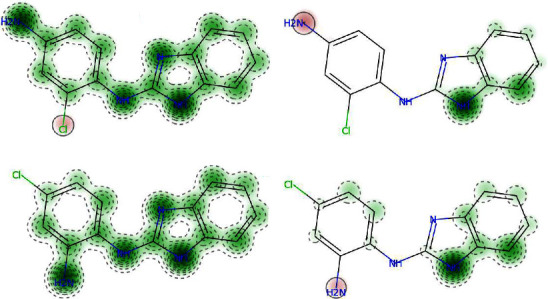
Acute inhalation toxicity of compounds **7** (a) and **8** (c) and skin sensitization of compounds **7** (b) and **8** (d).

In the development of new pharmaceuticals, the relationship between molecular structure, biological activity, and toxicity is of paramount importance. The presence of specific functional groups, rigidity of the structure, atomic arrangement, and interatomic radii influence the toxicity of the molecule [37]. The expected results were denoted by a negative sign (−) for nonharmful compounds and a positive sign (+) for toxic compounds [38]. The functional groups contributed to the toxicity characteristics were also anticipated (Table 2). The structural color green denotes the functional group that contributes to the nontoxic characteristics, whereas the structural color brown denotes the functional group that contributes to the toxic characteristics. The compounds **7** and **8** were found to be nontoxic for acute inhalation and sensitization of the epidermis. The chlorine atom and amine group (-NH_2_) were responsible for toxicity, as shown in Figure 7.

### 3.5. Molecular Docking Analysis

**Figure 8:**
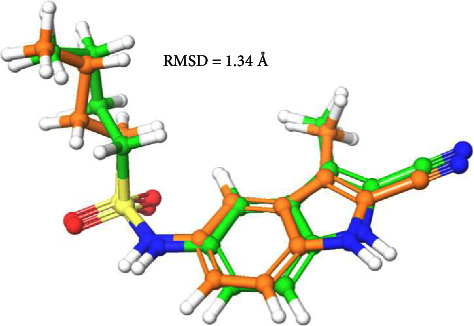
Validation of the molecular docking protocol employed using before docking (green) and after glide SP docking (orange) pose of cocrystallized ligand (GSK DG167).

Molecular docking studies have been carried out to inquire into the binding mechanism and binding energy of the synthesized compounds and to reveal the ligand-protein interactions behind the observed KasA selectivity [39]. The validation of the molecular docking method was conducted using the redocking approach, employing the cocrystallized ligand (GSK DG167) bound to the *Mtb* KasA protein. This involved docking the cocrystallized ligand within the binding pocket of the Mtb KasA protein and comparing the resulting docked pose with the crystal structure pose through the calculation of the root-mean-square deviation (RMSD) value, which was found to be 1.34 Å. As a general criterion, a docking method can be deemed valid if the RMSD value is ≤ 2.0 Å.  Figure 8 illustrates that the docked pose nearly perfectly overlapped with the crystal orientation of the Mtb KasA protein. This strongly suggests the validity of our docking method.

The binding affinity and mode of binding at the binding site of Mtb KasA (PDB ID: 6P9K) were investigated to gain a clearer understanding of the biological activities by which the synthesized benzimidazole analogs induced their efficacy [40]. Mtb KasA was chosen as the docking target because its crystal structure with a cocrystallized benzimidazole inhibitor was readily available [41]. In addition, it was reported that the benzimidazole-based inhibitors GSK DG167 and their analogs were investigated as KasA inhibitors. Benzimidazole-based inhibitors have demonstrated promising activity against this target, indicating that KasA is a validated drug target for Mtb. It was discovered that the nitrogen atom and bridge amine group on the imidazole moiety are advantageous, as are the substituted amine groups on the side ring connected to the benzimidazole ring. Compounds **7** and **8** had the highest docking scores of −7.368 and −7.173 kcal/mol, respectively. In Table 3, the binding scores of all compounds are listed.

**Figure 9:**
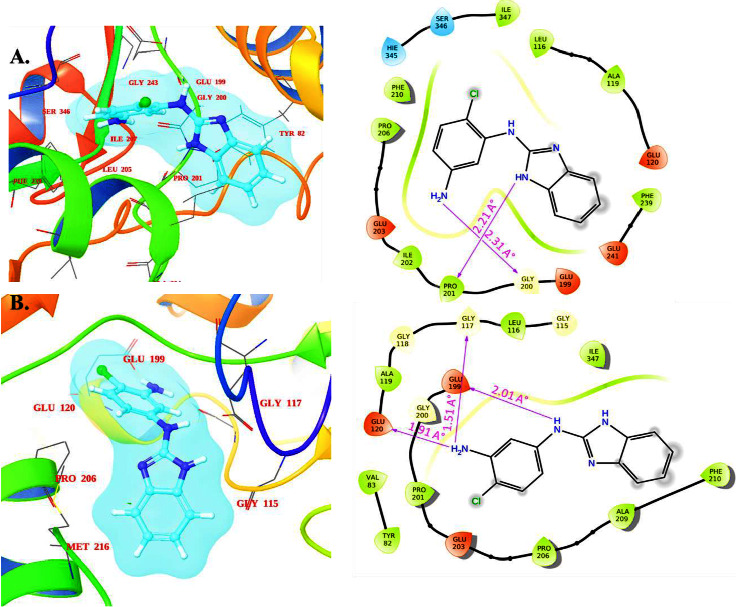
(a) 3D- and 2D-binding interaction of promising compound **7** and (b) 3D- and 2D-binding interaction of promising compound **8** in the active site of Mtb KasA protein (PDB ID: 6P9K).

As shown in Table 3, the synthesized compounds have a docking score between −5.149 and −7.541 kcal/mol when compared to the other cocrystallized ligand, DG167 (−7.101 kcal/mol). All the synthesized compounds **(1**–**12)** were found to fit snugly into the active site of the enzyme at sites near DG167, which is composed of the amino acids Glu 199, Glu 200, Pro 201, Ile 202, Glu 203, Pro 206, Phe 239, and Ile 347. MIC values of 0.8 *μ*g/mL indicate that compounds **7** and **8** exhibit promising inhibition in vitro. The docking scores of −7.368 and −7.173 kcal/mol for compounds **7** and **8** indicate that they can bind to the active site of KasA with significant binding affinity. The nitrogen of the p-amino phenyl ring and the benzimidazole moiety of compound **7** engage in hydrogen bond interactions at 2.31 and 2.20 Å, respectively, as depicted in Figure 9. The secondary amino group in compound **8** creates a hydrogen bond with the carbonyl of Glu 199 (bond distance: 2.01 Å). The 3-amino phenyl group also forms bivalent hydrogen bonds with Gly 117 and Glu 120. A similar docking scores and binding affinities of compounds **7** and **8** with DG167 indicate that the synthesized compounds have the potential to inhibit Mtb KasA efficiently.

### 3.6. Molecular Dynamics Simulation Study

The utilization of MD modeling has emerged as a prominent and indispensable tool in the field of protein-ligand research, enabling scientists to delve into the intricate nuances of protein structural stability and the intricate molecular patterns of interaction [42]. The conformational sampling of the ligand, protein backbone, and side-chain atoms are achieved through the implementation of a suitably extensive MD simulation [43]. Contrary to what one might expect, this work of writing provides a deep understanding of the complex dynamics of the solvated system by illuminating the many nonbonded interactions that occur inside it and the consequent energetics. The extraordinary flexibility of binding sites and the profound influence of loop flexibility are just two examples of the complex mechanisms that this topic involves. By circumventing the constraints inherent in docking studies, this approach effectively eludes the limitations previously encountered, thereby offering precise estimations of binding affinity [44]. To better comprehend the binding process and the complex's stability over time, MD analyses of the target protein-ligand complex were conducted. Using a variety of quantitative metrics, including root mean square deviation (RMSD), root-mean-square fluctuation (RMSF), radius of gyration (RGyration), and protein-ligand H bond interactions (Table 4), the dynamic behavior of the entire simulated system was exhaustively investigated.

By analyzing the root-mean-square deviation (RMSD) of the protein alpha carbon atoms about simulation time, the overall structural variations and conformational stability of each compound were determined. The RMSD indicates how the structure of the protein's atoms changed during the MD simulation. Low RMSD is a reliable indicator of a more stable system, as demonstrated by the simulation results. A wide range of oscillations in the RMSD graph, on the other hand, indicates that ligand binding to the target protein structure is unstable [45].

**Figure 10:**
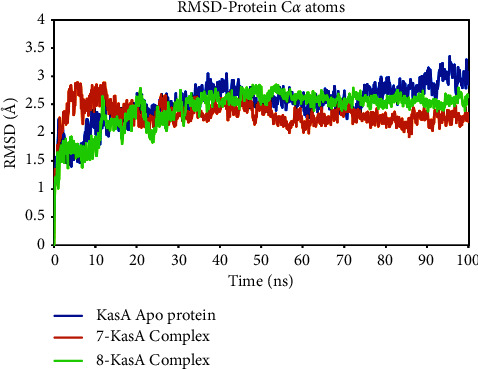
Time-dependent RMSD of C*α* atoms of Apo KasA protein and in complex with compounds **7** and **8**.

After the initial equilibration phase, the RMSD values of both the **7**-KasA and **8**-KasA complexes were stable within a range of 3 Å. The maximum RMSD deviation observed for the **7**-KasA complex was 2.89 Å, while the maximum RMSD deviation for the **8**-KasA complex was 2.86 Å. These results indicate that compounds **7** and **8** were able to bind and interact with KasA stably. The Apo KasA protein, on the other hand, exhibited a greater maximal fluctuation with a maximum RMSD deviation of 3.36 Å. This suggests that the Apo KasA protein underwent moderate structural changes and fluctuations during simulations in the absence of compounds **7** or **8** (Figure 10). The average RMSD values of 2.33 Å for the **7**-KasA complex and 2.42 Å for the **8**-KasA complex further support the stability of the complexes. The marginally lower average RMSD for the **7**-KasA complex compared to the **8**-KasA complex suggests that it may have attained a slightly more stable conformation. Overall, these results suggest that compounds **7** and **8** interacted effectively with KasA and formed stable complexes, as exemplified by the relatively low RMSD values and acceptable variations observed during simulations.

**Figure 11:**
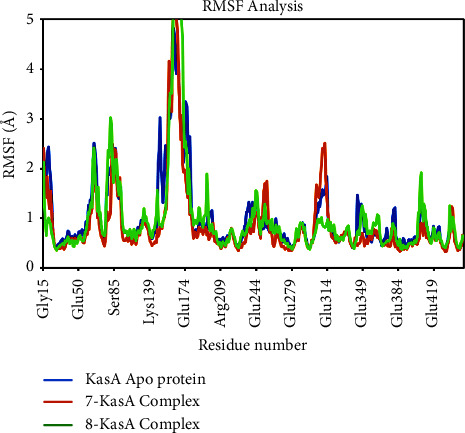
RMSF of individual amino acids of C*α* atoms of Apo KasA protein and in complex with compounds **7** and **8**.

The assessment of root-mean-square fluctuation (RMSF) offers a comprehensive portrayal of the dynamic tendencies exhibited by individual residues within the protein backbone. This evaluation considers their specific placement and participation in the interaction with a designated ligand. Through the meticulous examination of protein structures, this comprehensive analysis unveils the remarkable ability to discern regions of adaptability and flexibility. A discernibly elevated RMSF value serves as an indicator of an augmented degree of atomic fluctuation within the atomic C*α* coordinates of the protein, in contrast to its customary position as observed in the MD simulations [46]. The examination of the complexes revealed a striking similarity in the overall pattern of residue fluctuations. This remarkable consistency was vividly illustrated by the RMS plot, which showcased the nearly identical nature of these fluctuations. Except for a limited number of residues situated in the loop regions and C-terminal ends, which are distantly positioned from the site where the ligand binds, the majority of complexes displayed a moderate level of variation. Notably, key residues interacting with compounds **7** and **8** in the KasA binding site, such as Thr 114, Leu 116, Gly 117, Ala 119, Ile 122, Val 123, Glu 199, Gly 200, Pro 201, Ile 202, Glu 203, Leu 205, Pro 206, Phe 210, Phe 239, Gly 240, Ile 347, and Phe 404, displayed rigid behavior with minimal fluctuations (RMSF <1.5 Å) in MD simulation experiments (Figure 11). The low flexibility of these residues confirms their ability to form stable interactions with the promising compounds **7** and **8** compared to other residues in the KasA protein binding site.

**Figure 12:**
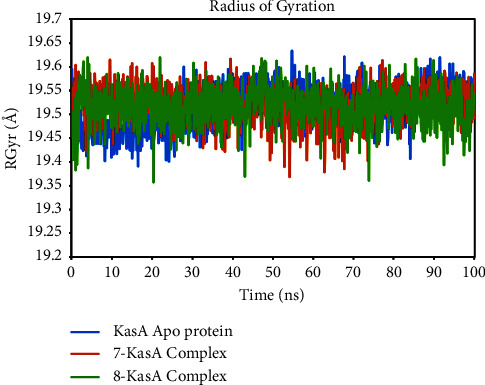
Time-dependent radius of gyration (RGyr) of KasA protein and in complex with compounds **7** and **8**.

The radius of gyration (RGyr) analysis was performed to investigate the compactness and folding pattern of the Apo KasA protein, as well as the ligand-bound complexes with compounds **7** and **8**. The RGyr values for the Apo KasA protein, **7**-KasA complex, and **8**-KasA complex are presented in Table 4. The results indicate that all three systems exhibited similar RGyr patterns, suggesting that the presence of ligands did not induce significant structural switching in the KasA protein. This observation further supports the stability of the protein-ligand complexes. The difference between maximum and minimum RGyr values observed was 0.23 Å for the Apo KasA protein, 0.23 Å for the **7**-KasA complex, and 0.26 Å for the **8**-KasA complex (Figure 12). These findings suggest that there were minimal differences in the overall compactness and folding patterns among the systems. Figure 11 provides a visual representation of the RGyr patterns for the free KasA protein and the ligand-bound KasA protein. The absence of prominent shifts or changes in the RGyr values further confirms the stability of the protein-ligand complexes.

**Figure 13:**
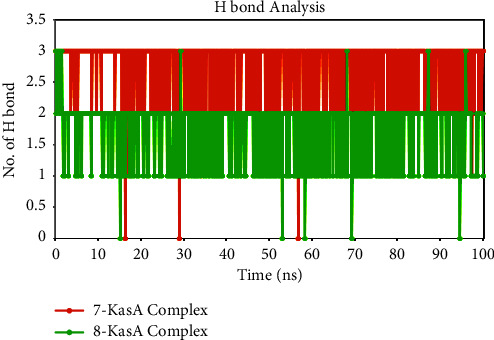
Time-dependent hydrogen bond analysis of KasA protein in complex with compounds **7** and **8**.

Hydrogen-bond analysis plays a crucial role in MD simulations for understanding molecular interactions and their dynamics. Hydrogen bonds are formed when a hydrogen atom interacts with an electronegative atom. They contribute to the stability and specificity of protein-ligand complexes and can affect binding affinities and conformational changes [47]. The **7**-KasA complex and **8**-KasA complex were analyzed for hydrogen bonds in the current study. The minimum number of hydrogen bonds detected in the **7**-KasA complex was 1, while the maximum was 3, with an average of 2.69. Similarly, the minimal and maximum numbers of hydrogen bonds for the **8**-KasA complex were 1 and 3, respectively, with an average of 1.72 (Figure 13). The analysis of hydrogen bonds reveals the presence of hydrogen bonds in both complexes, indicating the possibility of stabilizing interactions between the ligands and the KasA protein. The average number of hydrogen bonds provides an estimate of the overall frequency and strength of these interactions. The higher number of average hydrogen bonds in the **7**-KasA complex (2.69), compared to the **8**-KasA complex (1.72), suggests that compound **7** forms more hydrogen bonds with the KasA protein. This may indicate stronger and more extensive interactions between compound **7** and the protein, which may contribute to its increased binding affinity and site stability.

**Figure 14:**
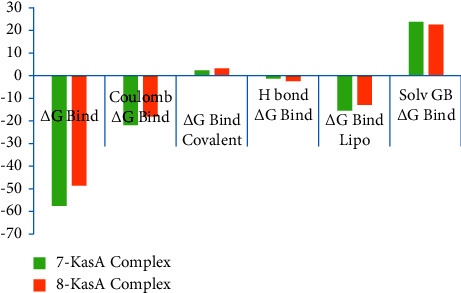
MM-GBSA-binding free energy components of **7**-KasA and **8**-KasA complexes.

The molecular mechanics/generalized born surface area (MM/GBSA) method serves as a pivotal tool in elucidating the energetics underlying molecular interactions postmolecular dynamics (MDs) simulations. Its significance lies in its ability to provide a detailed and comprehensive understanding of the binding free-energy landscape, offering valuable insights into the stability and affinity of molecular complexes. It enables a quantitative assessment of the binding affinity between molecules by decomposing the overall binding free energy into individual contributions, such as van der Waals, electrostatic, and solvation energies [48]. The final 10 nanoseconds snapshot of the simulation trajectory was utilized for the binding free-energy analysis. The MM/GBSA analysis provides comprehensive insights into the binding free energies of the **7**-KasA complex and the **8**-KasA complex (Supplementary file, Table S1 and S2). Both complexes exhibit favorable overall binding energies, with ΔG bind values of −57.889 kcal/mol and −48.591 kcal/mol, respectively. Electrostatic interactions (ΔG bind Coulomb) contribute significantly to the favorable binding energies in both cases, with values of −22.234 kcal/mol and −18.007 kcal/mol, respectively. Moreover, hydrogen bonding interactions (ΔG bind H bond) also play a crucial role, contributing −1.782 kcal/mol and −2.397 kcal/mol to the binding energies of the **7**-KasA complex and the **8**-KasA complex, respectively. In addition, lipophilic interactions (ΔG bind Lipo) further stabilize the complexes, with values of −15.88 kcal/mol and −13.01 kcal/mol, respectively. However, the positive contribution of solvation energy (ΔG bind Solv GB) suggests unfavorable solvation effects upon ligand binding, with values of 24.035 kcal/mol and 22.429 kcal/mol for the **7**-KasA complex and the **8**-KasA complex, respectively (Figure 14). Although covalent interactions (ΔG bind covalent) play a minor role, their negligible contribution indicates the absence of significant covalent bonding. Overall, these findings underscore the stable nature of both complexes, emphasizing the importance of electrostatic, hydrogen bonding, and lipophilic interactions in mediating ligand-receptor binding.

## 4. Conclusion

The purpose of this investigation was to conduct a comprehensive analysis of the antimycobacterial properties of a series of recently developed benzimidazole derivatives, specifically twelve compounds denoted as **1**–**12**. In addition, the study aimed to assess the intricate molecular interactions between these compounds and the KasA protein. The synthesized compounds underwent characterization using various spectral techniques, and their efficacy was investigated through in silico analysis. The remarkable inhibitory activity against the *M. tuberculosis* H37RV strain, with a MIC value of 0.8 *μ*g/mL, was observed in the chloro aniline-substituted benzimidazole derivatives, specifically compounds **7** and **8**. Moreover, a comprehensive investigation involving molecular docking and MD simulation analysis was conducted to validate the interaction between the KasA protein and the ligand. The results of this study revealed distinct hydrogen bonding patterns between the ligand and the protein, further supporting the binding affinity between the two entities. It is worth noting that compounds **7** and **8** exhibited the most favorable binding scores, measuring at −7.368 and −7.173 kcal/mol, respectively. Upon careful examination of the in vitro and in silico studies, one can confidently surmise that compounds **7** and **8** possess the inherent capability to emerge as the most promising inhibitors of Mtb.

## Figures and Tables

**Table 1:** The MIC value of synthesized compounds.

Abbreviation	MIC (*μ*g/mL)
**1**	25
**2**	50
**3**	1.6
**4**	6.25
**5**	6.25
**6**	1.6
**7**	0.8
**8**	0.8
**9**	1.6
**10**	6.25
**11**	6.25
**12**	6.25
Reference drugs	
Isoniazid	1.6
Ethambutol	1.6
Pyrazinamide	3.12
Rifampicin	0.8

**Table 2:** Acute inhalation toxicity and skin sensitization of compounds **7** and **8**.

Compound code	Acute inhalation toxicity	Skin sensitization
**7**	−	−
**8**	−	−

“+” = toxic, “−” = nontoxic.

**Table 3:** Glide docking score of synthesized compounds (**1**–**12**) in the active of Mtb KasA protein (PDB ID: 6P9K).

Compound code	Docking score
**1**	−5.149
**2**	−7.541
**3**	−5.869
**4**	−5.948
**5**	−6.557
**6**	−6.651
**7**	−7.368
**8**	−7.173
**9**	−6.331
**10**	−6.331
**11**	−6.143
**12**	−6.188
Cocrystal ligand	−7.101

**Table 4:** The minimum, maximum, and average values of different parameters, RMSD, RMSF, RGyr, and hydrogen bonding of the studied complexes.

	Root-mean-square deviation Å (RMSD)			Root-mean-square fluctuation Å (RMSF)			Radius of gyration (RGyr) Å			Hydrogen bonding		
	Min	Max	Avg	Min	Max	Avg	Min	Max	Avg	Min	Max	Avg
Apo KasA protein	1.09	3.36	2.53	0.37	4.83	0.99	19.39	19.62	19.54	—	—	—
**7**-KasA complex	2.22	2.89	2.33	0.33	5.39	0.87	19.37	19.60	19.53	1.00	3.00	2.69
**8**-KasA complex	0.84	2.86	2.42	0.36	8.41	1.00	19.36	19.62	19.51	1.00	3.00	1.72

Min: minimum, Max: maximum, Avg: average.

## Data Availability

The data used to support the findings of this study are available from the corresponding author upon request.

## Supplementary Materials

Supplementary Materials Table S1: MM-GBSA-binding free energies components for the 7-KasA complex obtained from molecular dynamics trajectories. Table S2: MM-GBSA-binding free energies components for the 8-KasA complex obtained from molecular dynamics trajectories. Figure S1: FTIR spectra of ST02. Figure S2: FTIR spectra of ST03. Figure S3: FTIR spectra of ST04. Figure S4: FTIR spectra of compound 1. Figure S5: 1H-NMR of compound 1. Figure S6: GCMS data with library search results for compound 1. Figure S7: FTIR spectra of compound 2. Figure S8: 1H-NMR of compound 2. Figure S9: FTIR spectra of compound 3. Figure S10: NMR spectra of compound 3. Figure S11: FTIR spectra of compound 4. Figure S12: 1H-NMR of compound 4. Figure S13: FTIR spectra of compound 5, Figure S14: 1H-NMR of compound 5, Figure S15: FTIR spectra of compound 6, Figure S16: 1H-NMR of compound 6. Figure S17: FTIR spectra of compound 7. Figure S18: 1H-NMR of compound 7. Figure S19: FTIR spectra of compound 8. Figure S20: 1H-NMR of compound 8. Figure S21: FTIR spectra of compound 9. Figure S22: 1H-NMR of compound 9. Figure S23: FTIR spectra of compound 10. Figure S24: 1H-NMR of compound 10. Figure S25: FTIR spectra of compound 11. Figure S26: 1H-NMR of compound 11. Figure S27: FTIR spectra of compound 12. Figure S28: 1H-NMR of compound 12.
