# Supplementary material for: Synthesis, Molecular Docking, Molecular Dynamic Simulation Studies, and Antitubercular Activity Evaluation of Substituted Benzimidazole Derivatives
Source: Adv Pharmacol Pharm Sci. 2024 Mar 28;2024:9986613. doi: 10.1155/2024/9986613 (PMC10994708; doi:10.1155/2024/9986613)
Supplement: Supplementary Materials — Table S1: MM-GBSA-binding free energies components for the 7-KasA complex obtained from molecular dynamics trajectories. Table S2: MM-GBSA-binding free energies components for the 8-KasA complex obtained from molecular dynamics trajectories. Figure S1: FTIR spectra of ST02. Figure S2: FTIR spectra of ST03. Figure S3: FTIR spectra of ST04. Figure S4: FTIR spectra of compound 1. Figure S5: 1H-NMR of compound 1. Figure S6: GCMS data with library search results for compound 1. Figure S7: FTIR spectra of compound 2. Figure S8: 1H-NMR of compound 2. Figure S9: FTIR spectra of compound 3. Figure S10: NMR spectra of compound 3. Figure S11: FTIR spectra of compound 4. Figure S12: 1H-NMR of compound 4. Figure S13: FTIR spectra of compound 5, Figure S14: 1H-NMR of compound 5, Figure S15: FTIR spectra of compound 6, Figure S16: 1H-NMR of compound 6. Figure S17: FTIR spectra of compound 7. Figure S18: 1H-NMR of compound 7. Figure S19: FTIR spectra of compound 8. Figure S20: 1H-NMR of compound 8. Figure S21: FTIR spectra of compound 9. Figure S22: 1H-NMR of compound 9. Figure S23: FTIR spectra of compound 10. Figure S24: 1H-NMR of compound 10. Figure S25: FTIR spectra of compound 11. Figure S26: 1H-NMR of compound 11. Figure S27: FTIR spectra of compound 12. Figure S28: 1H-NMR of compound 12. [file 9986613.f1.docx]

**Synthesis, molecular docking, molecular dynamic simulation studies, and anti-tubercular activity evaluation of substituted benzimidazole derivatives**

**Shankar Thapa^1,2,3*^, Mahalakshmi Suresha Biradar^2,4^, Shachindra L. Nargund^2^, Iqrar Ahmad^5^,** **Mohit Agrawal^6^**, **Harun Patel^7^, Ashish Lamsal^1^**

*^1^Department of Pharmacy, Universal College of Medical Sciences, Bhairahawa-32900, Nepal*

*^2^Department of Pharmaceutical Chemistry, Nargund College of Pharmacy, Bengaluru-560085, Karnataka, India*

*^3^Department of Pharmacy, Madan Bhandari Academy of Health Sciences, Hetauda, Nepal*

*^4^Department of Pharmaceutical Chemistry, Al-Ameen College of Pharmacy, Bengaluru-560027, Karnataka, India*

*^5^Department of Pharmaceutical Chemistry, Prof. Ravindra Nikam College of Pharmacy, Gondur, Dhule-424002, Maharashtra, India*

*^6^School of Medical & Allied Sciences, K.R. Mangalam University, Gurugram, Haryana, India,*

*^7^Division of Computer Aided Drug Design, Department of Pharmaceutical Chemistry, R. C. Patel Institute of Pharmaceutical Education and Research, Shirpur-425405, Maharashtra, India*

Correspondence should be addressed to Shankar Thapa; [tshankar551@gmail.com](mailto:tshankar551@gmail.com)

Table S1: MM-GBSA binding free energies components for the 7-KasA Complex obtained from molecular dynamics trajectories.

| **Frame No.** | **MMGBSA** | | | | | |
| --- | --- | --- | --- | --- | --- | --- |
|  | **ΔG Bind** | **ΔG Bind**  **Coulomb** | **ΔG Bind Covalent** | **ΔG Bind**  **H bond** | **ΔG Bind Lipo** | **ΔG Bind**  **Solv GB** |
| **900** | -56.611 | -22.888 | 3.924 | -1.600 | -15.502 | 23.471 |
| **901** | -56.870 | -22.669 | 5.197 | -2.008 | -16.498 | 22.341 |
| **902** | -57.801 | -22.691 | 2.602 | -1.545 | -15.322 | 23.465 |
| **903** | -59.513 | -24.178 | 3.801 | -1.934 | -16.663 | 25.238 |
| **904** | -55.057 | -18.916 | 3.017 | -1.803 | -15.783 | 23.689 |
| **905** | -59.782 | -23.695 | 1.809 | -1.769 | -15.911 | 25.906 |
| **906** | -59.859 | -21.400 | 1.801 | -1.931 | -16.374 | 25.238 |
| **907** | -58.263 | -22.887 | 1.811 | -1.961 | -14.702 | 23.993 |
| **908** | -56.809 | -19.277 | 1.031 | -1.394 | -16.339 | 22.719 |
| **909** | -56.517 | -21.418 | 2.649 | -1.621 | -16.133 | 22.821 |
| **1000** | -59.694 | -24.553 | 2.294 | -2.036 | -15.456 | 25.504 |
| **Maximum** | -59.859 | -24.553 | 1.031 | -2.036 | -16.663 | 22.341 |
| **Minimum** | -55.057 | -18.916 | 5.197 | -1.394 | -14.702 | 25.906 |
| **Average** | -57.889 | -22.234 | 2.721 | -1.782 | -15.880 | 24.035 |
| **STD (±)** | 1.651 | 1.835 | 1.200 | 0.215 | 0.595 | 1.241 |

Table S2**:** MM-GBSA binding free energies components for the 8-KasA Complex obtained from molecular dynamics trajectories.

| **Frame No.** | **MMGBSA** | | | | | |
| --- | --- | --- | --- | --- | --- | --- |
|  | **ΔG Bind** | **ΔG Bind**  **Coulomb** | **ΔG Bind Covalent** | **ΔG Bind**  **H bond** | **ΔG Bind Lipo** | **ΔG Bind**  **Solv GB** |
| **900** | -48.885 | -22.412 | 5.059 | -2.603 | -13.269 | 25.543 |
| **901** | -52.530 | -20.108 | 3.008 | -2.683 | -12.853 | 23.760 |
| **902** | -51.585 | -18.257 | 3.512 | -2.647 | -13.150 | 22.877 |
| **903** | -53.020 | -18.584 | 3.058 | -2.755 | -13.739 | 23.001 |
| **904** | -49.546 | -18.481 | 3.519 | -2.496 | -12.875 | 20.776 |
| **905** | -50.434 | -20.992 | 4.189 | -2.253 | -12.991 | 22.038 |
| **906** | -48.596 | -17.279 | 2.035 | -2.682 | -12.728 | 23.065 |
| **907** | -47.341 | -18.293 | 4.140 | -2.587 | -12.410 | 22.131 |
| **908** | -40.460 | -12.324 | 2.730 | -1.736 | -12.894 | 20.485 |
| **909** | -46.266 | -13.442 | 2.151 | -2.129 | -13.205 | 20.224 |
| **1000** | -45.840 | -17.908 | 2.931 | -1.799 | -13.000 | 22.823 |
| **Maximum** | -53.020 | -22.412 | 2.035 | -2.755 | -13.739 | 20.224 |
| **Minimum** | -40.460 | -12.324 | 5.059 | -1.736 | -12.410 | 25.543 |
| **Average** | -48.591 | -18.007 | 3.303 | -2.397 | -13.010 | 22.429 |
| **STD (±)** | 3.594 | 2.951 | 0.906 | 0.365 | 0.340 | 1.555 |

1. **IR graph of compound ST02 to ST04**

**Figure S1**: FTIR spectra of **ST02**.

**FT-IR range:** 3356 cm^-1^ (NH str primary amine), 2923 cm^-1^ (CH str aromatic), 3085 cm^-1^ (CH_3_ str), 1771 cm^-1^(C=O str carbonyl).

**Figure S2**: FTIR spectra of **ST03**.

**FT-IR range:** 3407 cm^-1^ (NH str secondary amine), 2923 cm^-1^ (CH str aromatic), 1628 cm^-1^(C=C str aromatic), 1511 cm^-1^(N=O str carbonyl).

**Figure S3**: FTIR spectra of **ST04**.

**FTIR Range:** 3494 and 3379 cm^-1^ (NH str secondary amine), 2924 cm^-1^ (CH str aromatic), 1642 cm^-1^(C=C str aromatic), 1503 cm^-1^(N=C str).

1. **IR and NMR spectra of synthesized substituted compounds (1-12).**

**Figure S4**: FT-IR spectra of compound **1.**


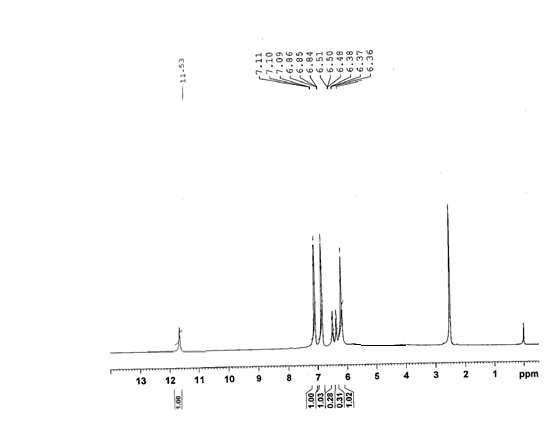

**Figure S5**: 1H-NMR of compound **1.**


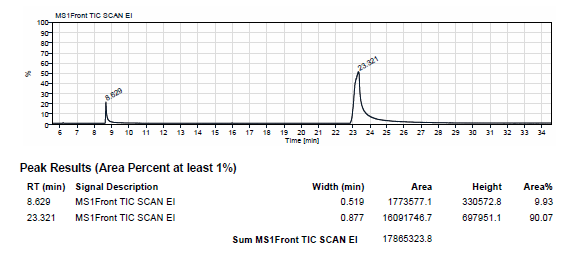


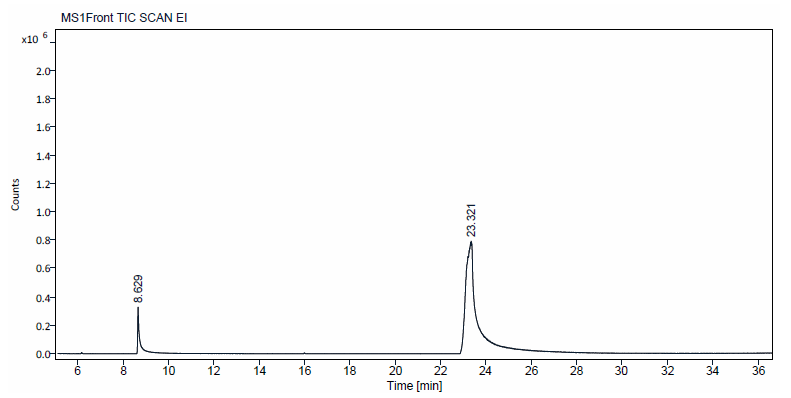


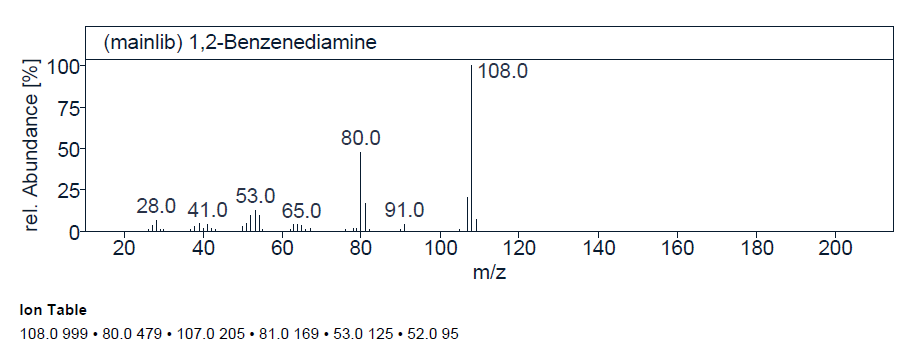


**Figure S6:** GCMS data with library search results for compound **1**.

**Figure S7**: FT-IR spectra of compound **2.**


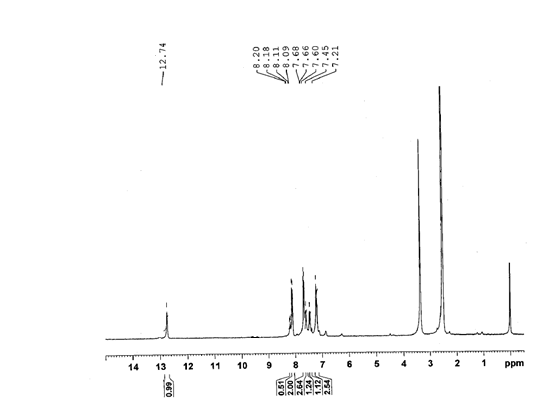

**Figure S8**: 1H-NMR of compound **2.**

**Figure S9**: FT-IR spectra of compound **3**.


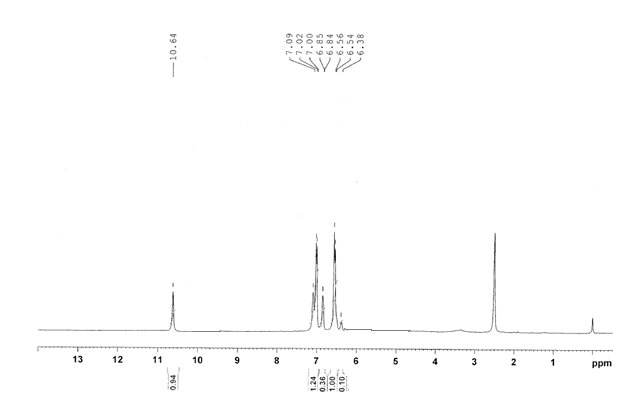

**Figure S10**: NMR spectra of compound **3.**

**Figure S11**: FT-IR spectra of compound **4**.


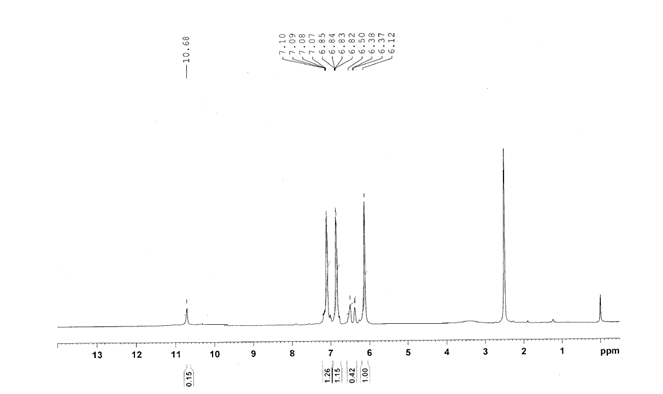

**Figure S12**: 1H-NMR of compound **4.**

**Figure S13**: FT-IR spectra of compound **5.**


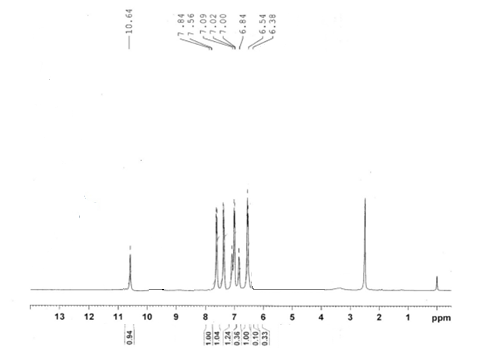

**Figure S14**: 1H-NMR of compound **5.**

**Figure S15**: FT-IR spectra of compound **6.**


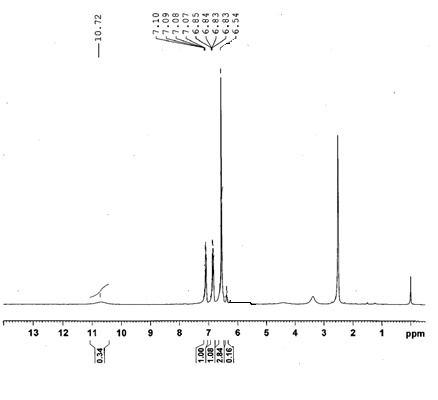

**Figure S16**: 1H-NMR of compound **6.**

**Figure S17**: FT-IR spectra of compound **7.**


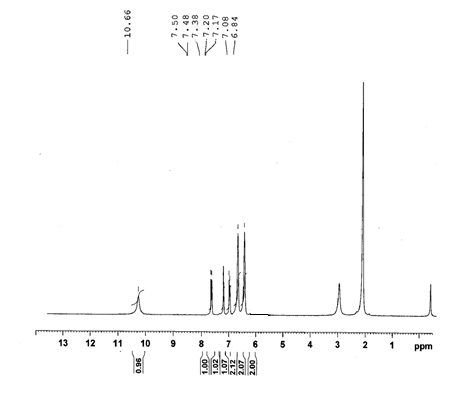

**Figure S18**: 1H-NMR of compound **7.**

**Figure S19**: FT-IR spectra of compound **8.**


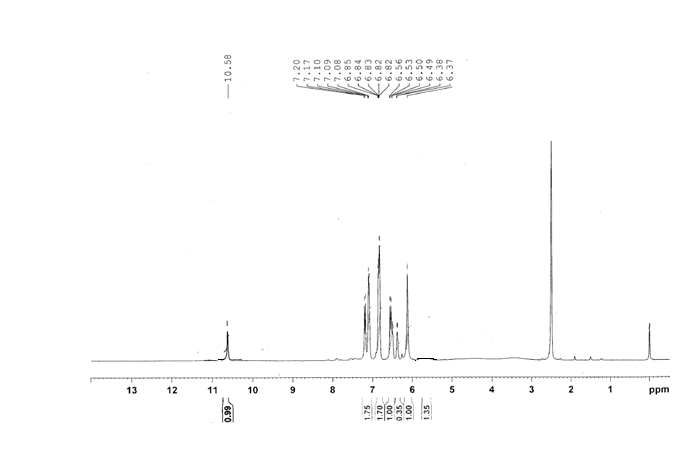

**Figure S20**: 1H-NMR of compound **8.**

**Figure S21**: FT-IR spectra of compound **9.**


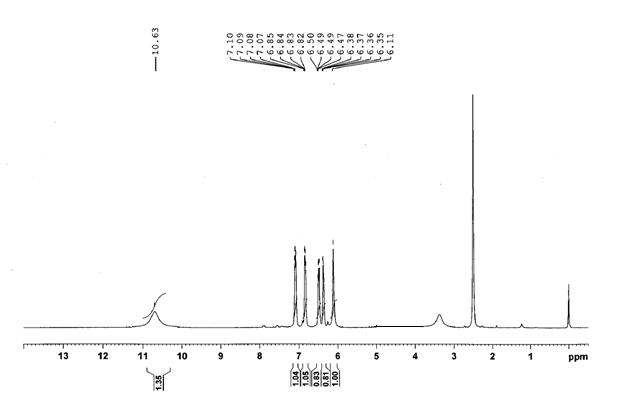

**Figure S22**: 1H-NMR of compound **9.**

**Figure S23**: FT-IR spectra of compound **10.**


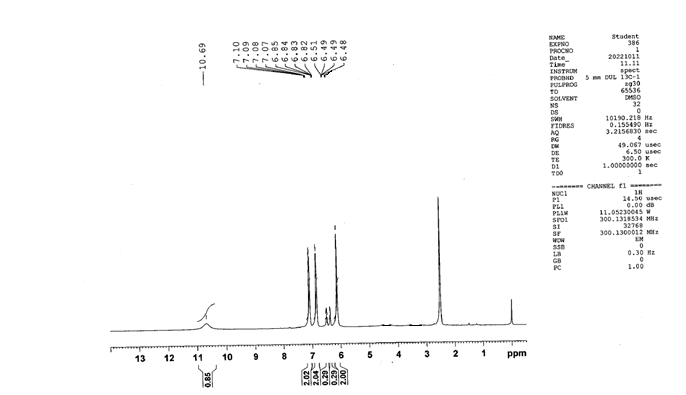

**Figure S24**: 1H-NMR of compound **10.**

**Figure S25**: FT-IR spectra of compound **11.**


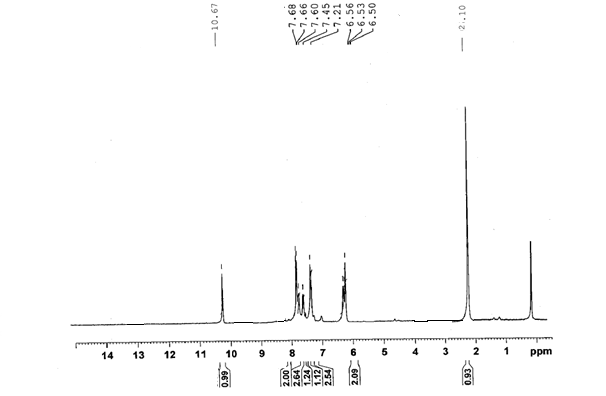

**Figure S26**: 1H-NMR of compound **11.**

**Figure S27**: FT-IR spectra of compound **12.**


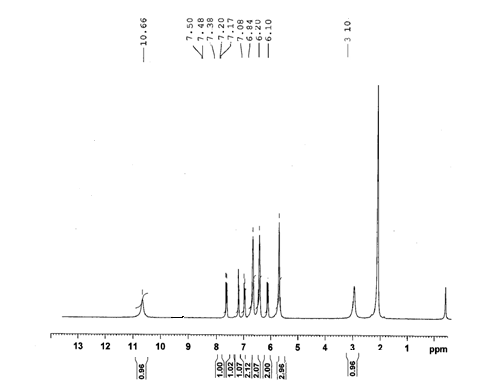

**Figure S28**: 1H-NMR of compound **12.**
